# Education that Empowers: A critical realist evaluation of a novel incivility-focused immersive simulation

**DOI:** 10.1186/s41077-026-00413-2

**Published:** 2026-03-19

**Authors:** Katherine Ralston, Valerie Rae, Ed Mellanby, Jane Hislop, Victoria Tallentire

**Affiliations:** 1 Medical Education Directorate, NHS Lothian, Little France Crescent, Old Dalkeith Road, Edinburgh, EH16 4SA UK; 2 Clinical Education, Edinburgh Medical School, University of Edinburgh, Edinburgh, United Kingdom; 3 NHS Education for Scotland, Edinburgh, United Kingdom

**Keywords:** Simulation, empowerment, civility

## Abstract

**Background:**

When we experience incivility, or rudeness, our performance suffers, negatively impacting patient safety. Immersive simulation-based education (SBE) has been under-explored as an educational strategy that may empower individuals to challenge incivility. Psychological empowerment was conceptualised by Spreitzer as four cognitive domains (meaning, competence, self-determination, and impact) that influence motivation and an active orientation towards work. We aimed to explore the mechanisms underlying the influence of incivility-based immersive SBE on the experiences of medical registrars when facing workplace incivility.

**Methods:**

We situated this study within a critical realist paradigm, a philosophical stance which aims to provide explanatory power to understanding the mechanisms that drive social reality. We conducted semi-structured interviews with 11 medical registrars at least four months after an incivility-based immersive simulation scenario. Interviews focused on participants’ experiences and reflections of workplace incivility, and the potential impact of their simulation experience. Data were analysed using template analysis, utilising Spreitzer’s theory of psychological empowerment as the guiding framework.

**Results:**

Incivility-based SBE stimulated powerful reflection relating to the cognitive domains of psychological empowerment. *Meaning* encompassed a sense that civility was important, with mechanisms of awareness and advocacy across boundaries. *Competence* referred to having the skills to manage incivility, with mechanisms of mindful response, self-regulation and perspective taking. *Self-determination* related to having autonomy when experiencing incivility, with mechanisms of identity formation and risk assessment. *Impact* was re-conceptualised as *transfer*, referring to the socio-structural mediators of transfer to practice, with mechanisms of socio-political support, participatory culture, and systems.

**Conclusions:**

We highlight the potential role of immersive SBE as a powerful tool to explore workplace incivility. SBE may influence behavioural change through provoking reflection related to psychological empowerment. We explore SBE as an influencer of the nuanced risk assessment that occurs when individuals face incivility. This framework of psychological empowerment has utility in designing effective SBE focused on relational aspects of work.

**Supplementary Information:**

The online version contains supplementary material available at 10.1186/s41077-026-00413-2.

## Background

Workplace incivility is common and has a devastating impact on the effective working of healthcare teams [1–4]. When we experience rudeness, our performance suffers, negatively impacting patient safety and staff wellbeing [4–6]. Education has a role in addressing workplace incivility [7], with simulation-based education (SBE) increasingly recognised as a modality that can influence professional relationships and workplace culture [8, 9]. Immersive SBE, in particular, is an under-explored educational technique that could have utility in empowering individuals to manage workplace incivility. If we can better understand SBE as a tool for shaping these relational aspects of work, we may improve our ability to design SBE that leads to behavioural and cultural change.

Incivility is rude or disrespectful behaviour that violates social norms for interaction in a given context, resulting in recipient distress [10, 11]. Incivility is driven by multiple factors, including personal stressors and a permissive workplace culture [12, 13]. When someone is rude, the performance of the entire team is impacted, threatening patient safety [5, 6, 14]. Incivility also contributes to staff burnout and attrition [15, 16]. Civility has therefore become a hot topic in healthcare, driven by movements such as ‘Civility Saves Lives’ [11] and prioritised by healthcare organisations and regulatory bodies [17, 18].

Although we are increasingly aware that the ways in which healthcare professionals interact has a profound impact on patient care and staff wellbeing, our understanding of how we can positively influence such interactions remains limited. Organisational interventions to improve working environments [19] or graded intervention programmes [20] clearly have a role in addressing incivility. Concerning an *individual’s* ability to challenge workplace incivility, the evidence for educational interventions is mixed. The literature predominately concentrates on interventions at the level of knowledge or skill rehearsal in low immersion environments [21]. Most educational interventions improve awareness of incivility, however evidence supporting subsequent behavioural change is limited [22–27]. Immersive SBE, a technique where learners interact with an authentic guided creation of an experience followed by debriefing, has remained unexplored in the incivility literature [21, 28]. It is important to acknowledge that SBE has been widely studied as an educational strategy to explore the distinct but related concept of “speaking up” behaviour, referring to a person being able to voice concerns, ideas or suggestions regarding work-related issues [29]. However, the speaking up literature primarily focuses on employee voice in the context of clinical patient safety concerns [30–32] rather than relational aspects of work, such as managing unprofessional behaviour [33]. The relationship between exposure to incivility and speaking up is complex, with one simulation study finding participants were more likely to speak up about patient safety when exposed to rudeness compared to civility [34]. Therefore, navigating workplace incivility is a distinct, and under-explored area within SBE.

Given that immersive SBE allows the exploration of performance-based outcomes realistic to the complexity of working in healthcare, this technique may have utility in empowering individuals to challenge incivility in practice [21, 28]. If we can better understand the ‘black box’ of immersive SBE focused on the navigation of professional interactions, we are more likely to be effective in the design, delivery and evaluation of SBE exploring relational aspects of work.

### Conceptual framework

**Fig. 1:**
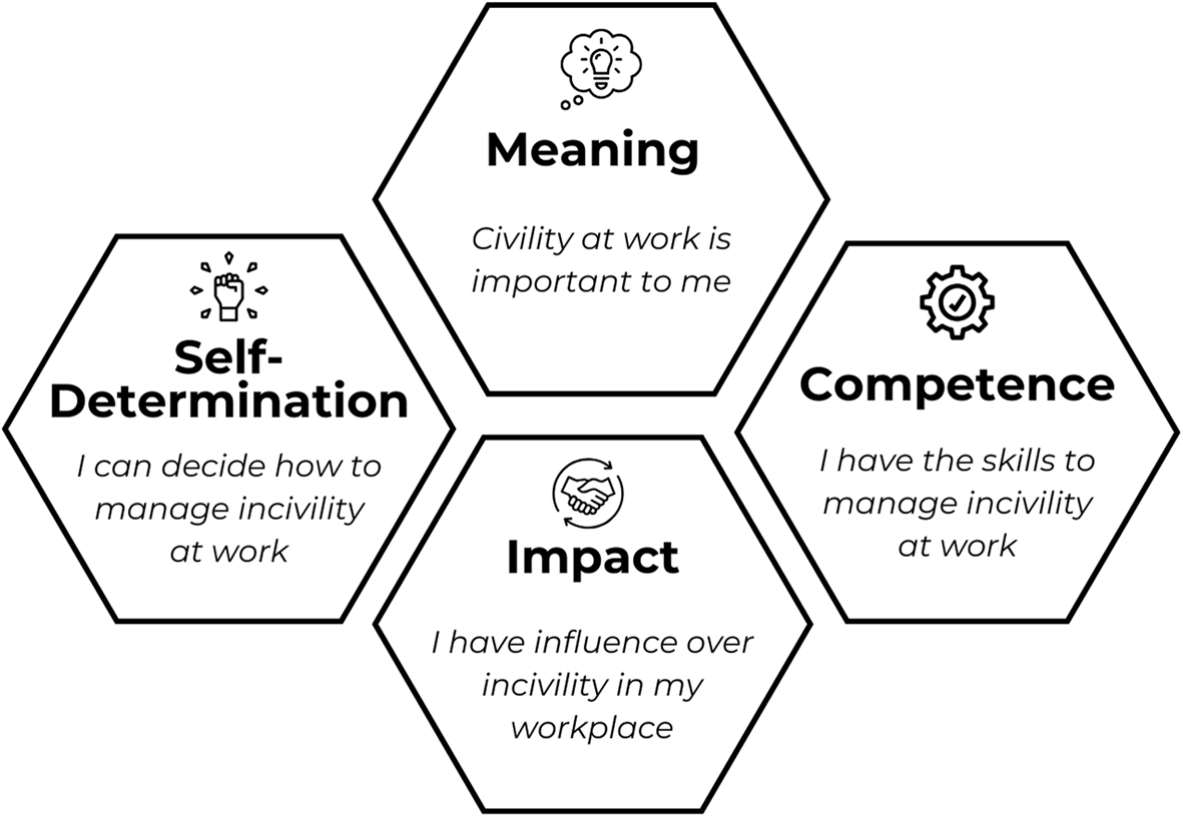
The four cognitions of individual psychological empowerment in the workplace described and validated by Spreitzer, as they relate to incivility [35]

When conceptualising this study, we were intrigued by empowerment theory as described within the business and organisational development literature [35, 36]. Empowerment holds relevance when considering the ability of an individual to navigate professional relationships and manage workplace incivility [37]. Psychological empowerment can be considered as a set of psychological states necessary for motivation and an active orientation towards one’s work [35]. Spreitzer described and validated a theory of individual psychological empowerment in the workplace, shaped by four cognitive domains: meaning; competence; self-determination; and impact [35] (Fig. 1). This was developed in the context of employees within the business sector, in order to validate a multidimensional measure of workplace psychological empowerment. Spreitzer warned against an overly individualistic concept of empowerment and advocated for an ecological approach that integrates the wider socio-structural context of the workplace, such as access to resources and workplace culture [36, 38, 39]. We utilised Spreitzer’s theory of psychological empowerment as a guiding conceptual framework for this study [35, 39].

### Philosophical orientation

In order to understand the mechanisms behind the influence of SBE, this study was situated within a critical realist paradigm [40]. Ontologically, critical realism considers that there is a reality that exists independent of our perception, which is driven by causal mechanisms [41]. Although we can extend our understanding of that reality through observation, our understanding will remain subjective and imperfect [42]. Epistemologically, critical realism aims to provide explanatory power in understanding the mechanisms that drive social reality [41]. This is relevant to exploring complex interventions such as SBE, to uncover “what works for whom, under what circumstances, how and why?” [43, 44]. Critical realism engages with existing theory but acknowledges that theory is fallible and can be supported, modified or rejected through analysis to provide the best explanation of reality [45].

**Fig. 2:**
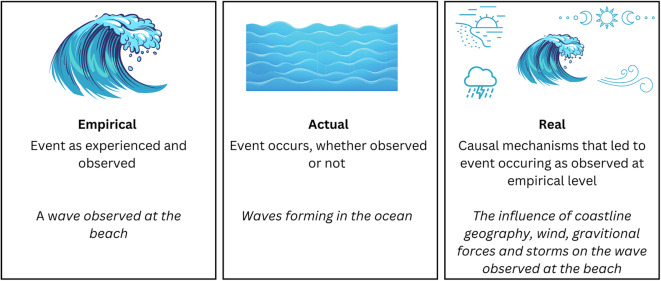
Illustrative example of levels of reality in critical realism

Critical realism uncovers the causal mechanisms that lead to events through considering reality at three levels: the empirical, actual, and real [45]. Empirical refers to events as we experience them. Actual refers to events occurring, whether we observe them or not. Real refers to the causal mechanisms influencing events. These concepts are detailed in Fig. 2 with an illustrative example. A critical realist lens allows us to iteratively build explanations for the outcomes of educational interventions, diving below the surface question of “does it work?” to examine “*why* and *in what ways* does it work?” [43].

### Aim

This study aimed to explore the mechanisms underlying the influence of incivility-focused immersive SBE on the experiences of medical registrars when facing workplace incivility.

## Methods

### Study design

The use of theory to inform study design resonates with the iterative explanation-building process underpinning critical realism [46]. However, this is with the understanding that theories must be critically examined during the analysis [40, 45]. In this case, we utilised and critiqued Spreitzer’s theory of psychological empowerment in the workplace [35].

### Context

This study was conducted in NHS Lothian, within South-East Scotland. Following their primary medical qualification, doctors in Scotland undertake two years of foundation training before starting specialty training. Those pursuing a medical specialty complete stage one of internal medicine training (IMT), followed by stage two of IMT during which they are commonly known as ‘medical registrars’.

This study focused on a one-day immersive simulation course to which all medical registrars within South-East Scotland were invited to voluntarily participate in via email. The course is centred around human factors and behavioural skills, with intended learning outcomes (ILOs) based on the IMT stage 2 curriculum [43], the General Medical Council Generic Professional Capabilities Framework [44] and findings from a study examining the influencers of patient safety in hospital from the perspective of medical registrars [45]. An immersive simulation scenario focused on incivility was developed as part of this new course, which involved the participant witnessing rudeness between members of the medical team in a non-clinical area (Additional File 1). The participant was expected to explore and challenge the incivil interaction, uncover the factors that have led to rudeness, and support the team in managing these factors and resolving team conflict. Participants were informed of the over-arching goals of the simulation course within the pre-briefing, but in accordance with usual practice at this centre, were not made aware of specific ILOs prior to each scenario. A maximum of six participants were included per course, with one participant leading this scenario while other participants observed via video. As per usual debriefing practice with this centre, simulation debriefing involved all participants and was completed by a trained facilitator using the generic Scottish Centre Debrief Model [47], with brief optional debriefing prompts reflecting ILOs included within the scenario guide (Additional File 1).

### Data collection and analysis

Using a convenience sampling method, all participants (*n* = 21, medical registrars across four course dates) who attended this simulation course between 1st March 2023 and 8th December 2023 were invited by email to participate in this research. Those who responded within six weeks of email invitation and gave consent were invited to an online interview between three and 12 months after the course. This follow-up time was to allow participants a period of reflection following the course and time during which they may encounter workplace incivility. The time gap between SBE and the interview varied between participants, primarily due to the timing of the simulation courses as well as availability of interviewer and participant. We also collected routine evaluation data from all course participants via an anonymous post-course electronic survey.

We developed a semi-structured interview schedule focused on exploring participants’ experiences of workplace incivility (Additional File 2). A critical realist approach aims to investigate participants’ reactions to the opportunities provided by the intervention to explore underlying mechanisms. Therefore, interview questions focused on how the session had influenced the participant’s views, understanding, or behaviours [43]. An abbreviated version of the interview schedule was shared with participants ahead of the interview to allow time to consider their responses. To encourage free and honest conversation, interviews were conducted by VR, who had no prior relationship with the participants. The interview schedule was discussed between VR and KR after the initial set of interviews and was felt to be effective in collecting the required data without significant further alteration. Due to the diverse location of participants across South-East Scotland, interviews were conducted online and scheduled according to participant availability. Interviews were audio-recorded, transcribed verbatim and analysed. Data were stored securely.

We analysed the data using template analysis, a flexible deductive approach underpinned by theory- and researcher-driven analysis compatible with a critical realist stance [45, 48]. Analysis was completed in parallel with data collection. The research team was instructed to code excerpts that related to the mechanisms underlying the influence of SBE on participants’ beliefs, reflections and experiences of incivility during and after the simulation intervention. Through this, we aimed to bring our critical realist lens to explore the causal mechanisms behind our educational intervention: the ‘why’ and ‘in what ways’ SBE works or does not work. KR analysed an initial data subset (n = 2), with ‘a priori’ codes based on Spreitzer’s theory of psychological empowerment in the workplace, as well as developing new and modified codes to form an initial coding template. These took the form of mechanisms relating to domains of psychological empowerment. Subsets of the data were then shared equally amongst the research team, with each transcript dual-coded by KR and another member of the team. Iterative modification and development of the coding template was driven by independent reflection, individual feedback, and collaborative discussion amongst the research team. This continued with data subsets until all transcripts were dual-coded and a final coding template had been agreed. Although detailed consideration was afforded to all team members views during this process, KR held final decision-making power regarding coding decisions. The final template was reviewed independently by JH and subsequently discussed between JH, KR and VT to ensure that themes best reflected the mechanisms underlying SBE. The final coding template was agreed by all authors and applied to the full data set by KR.

### Reflexivity

There was purposeful diversity within the research team. KR is a medical registrar with medical education research experience, who developed the simulation course and held prior interest in incivility from previous research as well as formative experiences of incivility in the workplace. VT is an acute medicine consultant with significant qualitative research and simulation experience. EM is an anaesthetist with experience in socio-cognitive skill training and research. VR is a paediatric doctor and medical education fellow. JH is a physiotherapist and academic in post-graduate medical education. VT and EM provided peer review of the incivility simulation scenario but were not involved in course delivery. The research team was purposely composed of those both involved (KR, VT, EM) and not involved (VR, JH) in the design of the educational intervention to encourage critical reflection. KR led the research team, keeping a reflective account on the research process and field notes within iterative versions of coding templates. It was agreed between the authors that KR held final decision-making power over methods and final wording, however frequent meetings and email discussions between the research team throughout data collection and analysis encouraged a move from the individual to the collective through collaboration, challenge, negotiation and reflection. Interpretation of findings is likely to have been influenced by KR’s individual views regarding the importance of addressing incivility in healthcare as well as all authors holding beliefs from previous work that SBE is a valuable educational technique. Previous work by KR and VT exploring the workplace experiences of medical registrars [49, 50] is also likely to have shaped our interpretations.

## Results

Within routine post-course evaluation data (*n* = 17), all participants would recommend the SBE course to a colleague, and comments related to incivility featured in 65% of participants key learning points over the entire course. Between March and April 2024, interviews were conducted with 11 medical registrars. These took place, on average, eight months following the simulation course (range three to 12 months). Interviews lasted an average of 31 min. Themes were identified relating to the cognitive domains of psychological empowerment in the workplace; meaning, competence and self-determination, with impact reconceptualised as transfer. The underlying mechanisms of SBE were explored within each theme, as discussed below and summarised in Table 1.

**Table 1:** Summary table of themes, mechanisms and illustrative quotes

Mechanism		Description of Mechanism		Illustrative Quote
**Meaning**				
Advocacy across boundaries		A sense of advocacy across boundaries influenced by observing incivility within an interprofessional team in the simulation followed by individual and peer reflection. This appeared to lead to a shift in values away from ‘protection’ of own professional group or team, and towards advocating for all colleagues.		*“It’s not just my responsibility to make sure the SHO [resident doctor] is okay…both sides*,* I have to make sure it’s okay. Whether it’s the FY1 or the radiographer*,* or whether it’s me and the radiographer*,* I don’t want the radiographer to have had rudeness or feel that they’ve been let down*,* in some way” (P7)*
Awareness		Greater awareness of the negative impacts of incivility through scenario observation and peer reflection on own and others’ experiences, standards and beliefs within the debriefing.		*“having a day like that with a focus on civility has helped my awareness of that*,* an awareness of the problems that incivility can cause for patient care and just wellbeing at work” (P4)*
**Competence**				
Mindful response		Consideration of the benefits of a ‘mindful’ response through observing and reflecting on different approaches to managing incivility, both within the simulation and through discussing real-world experiences with peers.		*“I think again I learnt from the scenarios to take a break and take a pause from things*,* because I think sometimes when you’re continually in a situation trying to come to an agreement it’s good for both sides to maybe take a pause and reflect back on what’s happening” (P3)*
Self-regulation		Deepened understanding of the impact of incivility on self, the factors that may lead to own incivility and how these factors may be managed, through engaging with the simulation scenario and reflection with peers.		*“if I reflect on times I’ve ever been incivil to people*,* it’s often when you’re working within a really stressed or pressed system*,* or there’s something else going on” (P9)*
Perspective taking		Greater recognition of the power of empathy and understanding others’ perspectives when managing incivility through engaging with the simulation scenario and reflection with peers.		*“the fact that it [the scenario] was largely you know discussing I think about how you speak to both parties to try and understand their views. But to not sort of take sides…trying to facilitate a discussion between everybody to make each other realise or recognise the pressures and stresses that each other are under but also to understand how their actions and behaviours might have been perceived to be unpleasant for others to experience” (P5)*
**Self-determination**				
Identity Formation		Development of a deeper understanding of the importance of human factors and behavioural skills when embodying a senior leadership role as the medical registrar, through experiencing the simulation scenario, debriefing with peers and individual reflection. This appeared to influence a perception of self as a leader and influencer within the system.		*“it [the scenario] definitely made it really clear that your role isn’t just about the patient management and the medical side of things*,* it’s the management of the team and managing personalities sometimes*,* or clashes. And that actually*,* for the safety and the good care of the patients*,* that you need to be cohesive as a team and work well together. And it’s not about your medical management*,* necessarily*,* it’s also about all the other non-technical aspects of the job” (P10)*
Risk Assessment		Development of a more nuanced sense of risk assessment when managing incivility through reflecting on the benefits of challenging incivility balanced with the risks to self or professional relationships.		*“as you become a wee bit more senior you probably feel a bit more responsibility for your team and the people that are around you…from that perspective I probably am more likely to try and discuss situations and events if there are things that have… that maybe have an incivility element to them” (P5)*
**Transfer**				
Socio-political support		The values, customs and beliefs of the team, department or organisation that influence incivility in the workplace.		*“when there’s just quite an antagonistic culture on the ward…seeing your team members are behaving uncivilly to other staff members*,* that cascades because everyone feels that’s an acceptable way to behave” (P2)*
Participatory Culture		The influence of sense of belonging, hierarchy and psychological safety on an individual’s perception of feeling valued and having a voice in an organisation		*“it is helpful to know that you are working in an environment where you are respected enough*,* and you are valued enough*,* that people would understand or recognise if you had an issue with something…and you would be supported to follow through with escalating that” (P6)*
Systems		The influence of organisational systems and workplace resources on the experience of incivility in the workplace		*“I think in terms of system change*,* very*,* very clear clinical pathways*,* referral criteria*,* communication pathways*,* I think*,* really does*,* when they are not in place creates a lot of stress*,* and with stress comes*,* sometimes*,* sadly*,* actions of incivility” (P9)*

### Meaning

The theme of meaning concerned the mechanisms through which the simulation scenario influenced the value participants place on addressing incivility in the workplace: namely that “incivility is important to me”.

The simulation appeared to prompt a greater awareness of the negative impacts of incivility through observing the scenario and engaging in peer reflection regarding own and others’ experiences and beliefs within the debriefing: “*I also learnt a lot from just listening in the reflections of other colleagues in the scenarios of what they’ve been involved with and how they’ve navigated that positively or negatively” (P3).* A deeper understanding of the impact of rudeness on individuals, teams, and patients appeared to enhance a sense of meaning regarding the importance of addressing incivility:

*“highlighting how detrimental incivility…can be to all members of the*,* all people that are involved*,* even the people just witnessing it…I think that was why it was a really powerful scenario because it was something that we could all relate to so easily” (P10)*.

Observing and debriefing a scenario that centred around incivility within an interprofessional team also prompted reflection regarding the importance of advocating for all colleagues regardless of profession or team. One participant reflected that within the scenario: *“my automatic feeling was to very much be my SHO’s [resident doctors] advocate and I think one of the things that I reflected on was that the coordinator [nursing colleague] also was very stressed…” (P7).* Witnessing a doctor being rude to a nursing colleague appeared to lead to a shift in values away from ‘protection’ of own professional group or team, and towards an expectation of a consistent standard of behaviour regardless of professional group or context:

*“I think you won’t be respected as being in a management role…[if you are] siding with where you feel familiar or where you feel your professional colleagues are*,* rather than actually looking and taking a step back and trying to understand different viewpoints… that’s probably been more of something I’ve taken from the scenario” (P3)*.

In summary, experiencing incivility-focused SBE appeared to increase the value participants place on maintaining workplace civility, through enhancing a sense of awareness of the detrimental impact of incivility as well as encouraging advocacy across professional boundaries.

### Competence

The theme of competence referred to the mechanisms behind the influence of the simulation scenario on individuals perceived skills in challenging incivility, or maintaining their own civility, at work.

Participants reflected on the benefits of taking a more mindful, balanced approach when responding to incivility, from *“[being] a bit gung-ho” (P1)* towards a *“pause and reflect” (P3)* or “*more measured approach*” *(P1).* This was achieved through observing and reflecting on different possible approaches, both within the simulation and through discussing real-world experiences with peers:

*“I quite liked the way that the colleague doing the scenario*,* I suppose didn’t immediately try and go in and just fix things. She probably thought about*,* take a pause…is there anything else going on that’s making this more intense or more emotionally charged?” (P3)*.

In addition, the simulation scenario also appeared to prompt greater recognition of the power of empathy and understanding other’s perspectives when managing incivility. This included the consideration that “*if someone’s being rude… actually*,* are they okay?” (P1)* and “*exploring the underlying issues that might make a person act or behave out of character*” *(P6).* Participants consistently identified that the ability to explore the perspectives of each party was essential to the effective management of incivility:

*“I think we often forget that…everybody has their own competing agendas and we’re not always very good at stopping and saying*,* ‘how’s your shift going now?’…and I think again with this scenario that’s probably one thing to think about is understanding different points of view” (P3)*.

In addition, experiencing the simulation scenario and debriefing prompted reflection from participants on the impact of incivility on themselves, as well as identifying and managing factors that may lead to their own rudeness. This led to consideration of how participants could self-regulate their own risk of incivility:

*“It’s [the scenario] made me reflect a bit more on myself…when you’re busy and how you communicate to people*,* that you can be a bit more aware yourself*,* of what you say to people” (P2)*.

In summary, experiencing incivility-focused SBE appeared to influence skills in managing incivility through the development of effective response strategies and improved self-regulation.

### Self-Determination

The theme of self-determination considered the mechanisms underlying the influence of the simulation scenario on participants sense of control or autonomy in initiating or regulating actions related to managing incivility or maintaining their own civility in the workplace.

Experiencing the scenario and engaging in the rare opportunity to debrief with peers as medical registrars stimulated reflection on the importance of behavioural skills within this role. This appeared to influence participants sense of identity as a leader and influencer within the system:

*“Since that scenario*,* and thinking about different points of view and thinking about my role as a medical registrar…how I see myself and view my role*,* has probably changed because it’s more of a leadership role… I’ve had to add to my armoury or whatever of not just being able to survive it for myself but trying to help other people work…through a difficult scenario or a situation (P7)*.

A further mechanism within SBE that influenced participants sense of control over addressing incivility was the development of a more nuanced sense of risk assessment, through reflecting on the benefits of challenging incivility balanced with the risks to self or professional relationships. Participants reflected on fears regarding “*escalating a situation*” *(P3)*, a sense of futility or a risk of damaging relationships. Participants also considered risk to self, whether emotional or related to career progression: “*you don’t want to create bad relationships…don’t want to impact your ability to get good training*” *(P5).*

However, developing the skills to manage incivility (competence) and understanding the detrimental impact of rudeness (meaning) through experiencing incivility-related SBE appeared to ameliorate these perceived risks: *“I think definitely now*,* knowing the impact of it I would definitely be more assertive in saying that I don’t think this is helping*,* let’s stop” (P10).* This also appeared to enhance a sense of duty to others in addressing incivility through identity formation as a medical leader: *“as you get more senior*,* you do just feel that you have that responsibility” (P5).*

In summary, engaging with incivility-focused SBE with peers appeared to contribute to professional identity development within the medical registrar role, with subsequent influence on the complex risk assessment that occurs when considering whether to challenge incivility.

### Transfer

The final element of Spreitzer’s psychological empowerment theory comprised ‘impact’; referring to the degree to which an individual can influence outcomes at work. We found that SBE had limited influence in this area, therefore re-imagined the conceptualisation of ‘impact’ to become ‘transfer’. The theme of transfer explores the socio-structural factors that can facilitate or hinder the transfer from SBE to practice. Through this, we could better understand the mechanisms behind *why* SBE may be successful or unsuccessful in influencing behaviour.

The values, customs and beliefs of a participant’s team, department or organisation influenced the ability for SBE to facilitate behavioural change:

“*the workplace has a culture…one day thinking differently in a simulated scenario is useful*,* but… it’s very easy to revert back to old patterns when you’re back in the usual environment”* (P4).

An acceptance for incivility within a team can lead to a sense of inertia in challenging rudeness: “*perhaps it’s known in the department that this is how someone behaves and clearly people have been aware of it…saying something’s not really going to change anything” (P2).*

However, participants repeatedly expressed that education regarding incivility, including within SBE, bolstered a sense of socio-political support for civility at work:

*“it did lead to conversations afterwards…my husband’s also a*,* well he’s a surgeon…I went home and told him all about the incivility…so I think it has led to conversations within the sim day and between those of us who were there. But then also going back and*,* kind of*,* telling our teams or our partners or our friends about it as well*,* so I think it’s a good way to disseminate that message” (P10)*.

The degree to which participants felt valued and had a voice within their team or organisation also influenced the ability to transfer learning into practice. This was influenced by a sense of belonging, with rotational jobs and lack of continuity perpetuating a feeling of vulnerability: “*a transient entity who doesn’t really belong” (P2).* Hierarchy was reflected as “*a big part of the challenge*” *(P5)* to speaking up, however relationship-building influenced a sense of voice: “*if the department is making some sort of effort to see you as an individual…I am no longer really just a number” (P6).* A sense of psychological safety, or feeling able to speak up without fear of consequence, was described as important: *“teams like that where everyone feels really empowered…you wouldn’t mind saying*,* oh*,* that made me feel a bit uncomfortable” (P2)*.

Finally, the systems within which participants work was cited as an important factor influencing the ability of SBE to lead to change, given that incivility is often perpetuated by poorly designed or under-resourced systems:

*“should we be focusing instead on the systems that create an environment in which incivility bubbles and brews…if I think about times I’ve been incivil*,* it’s because I’ve been so busy and stressed*,* and so if you cut half my workload out*,* it probably wouldn’t have happened?” (P9)*.

In summary, socio-structural mechanisms of socio-political support, participatory culture and systems can both help and hinder transfer of learning from SBE to the workplace. Interestingly, simply experiencing an incivility-based SBE increased the perception of socio-political support for civility amongst participants.

### Experiences of incivility after SBE

Many participants commented on how the SBE influenced subsequent concrete experiences of incivility following the course, as reflected in the preceding results. The majority of these experiences related to taking a more measured approach when managing incivility, reflecting on and reducing factors that could lead to participants own incivility, and improved perspective taking, advocacy and support for all team members. An example is detailed below, which relates to the theme of meaning:

*“One of my peers…was I suppose…being quite derogatory to the allied health professionals involved in the email…[participant asked if or how the simulation scenario influenced their experience of this event] I suppose*,* like it’s about recognizing that like that’s not appropriate… being aware quite early on that there’s some things going on that you don’t like…it comes back to the thing about having the kind of confidence and responsibility to think that that’s my, like should be everybody’s duty to be able to call that out” (P5)*.

Some participants reflected that it could be challenging to tease out influence specific to SBE compared to influence from other workplace, personal or educational experiences. Barriers cited to challenging incivility in practice included risk to self, time, private space, or conversations over the telephone or email.

## Discussion

In this study, we explore immersive SBE as a technique in overcoming the challenge of empowering healthcare professionals to manage workplace incivility. Utilising a critical realist lens, we explore the underlying mechanisms behind the impact of incivility-focused SBE through the use of psychological empowerment as a conceptual framework. We found that SBE can stimulate powerful reflection encompassing different cognitive domains of psychological empowerment in the workplace. Through these reflections, it is possible that SBE influenced a subsequent sense of psychological empowerment when managing incivility in practice.

There has been understandable hesitation in the use of immersive SBE to explore workplace relationships in areas such as incivility or microaggressions, with concerns including risk of participant or faculty distress and inadvertent development of unconscious bias [51–53]. Here, we demonstrate the feasibility and impact of immersive SBE with an *explicit and exclusive* focus on workplace relationships, generating powerful peer reflection that could influence behavioural change in areas such as self-regulation and perspective-taking. We would caveat this with highlighting the importance of authentic but thoughtful scenario design and compassionate facilitation. For example, we designed our scenario around outcomes that went beyond simply challenging incivility to exploring why incivility occurred, with a focus on supporting colleagues and conflict resolution. We felt that exposing participants to direct rudeness risked psychological distress, therefore deliberately ensured participant exposure to incivility was in the role of a witness. We contained incivility within the participant’s own team to reduce the risk of stereotyping other professional groups. The use of SBE as a potentially powerful tool to shape professional relationships and culture is reflected in the literature, although these important outcomes can often be hidden within clinically focused scenarios [9, 54, 55]. Translational simulation programmes in trauma and obstetric settings have been found to profoundly influence relational aspects of work, such as the concept of mutual respect [9, 54]. We encourage simulation educators to move forward to the deliberate and thoughtful incorporation of navigating professional relationships as a *central focus* within the objectives of simulation activities.

Within the theme of self-determination, the concept of risk assessment held particular interest; shedding light on the inherent and complex tensions that exist when individuals must decide whether to challenge incivility. The barriers to challenging incivility cited in the literature are broadly reflected in our findings, including fear of escalation or reprisal, hierarchy, lack of staff continuity, silo working, and workplace culture [56]. A novel finding within this study was a sense of responsibility towards others as a driver towards intervention when incivility occurs. This is distinct from previous sociological literature exploring incivility in the public sphere, where witnessing incivility commonly resulted in an ‘indifferent” response in comparison to direct incivility leading to an “intervention” response [57]. This is likely mediated by several factors, such as a sense of duty to the wider team that appeared to be heightened through reflection within SBE on the concepts of awareness, advocacy across boundaries and identity formation as a medical leader.

**Fig. 3:**
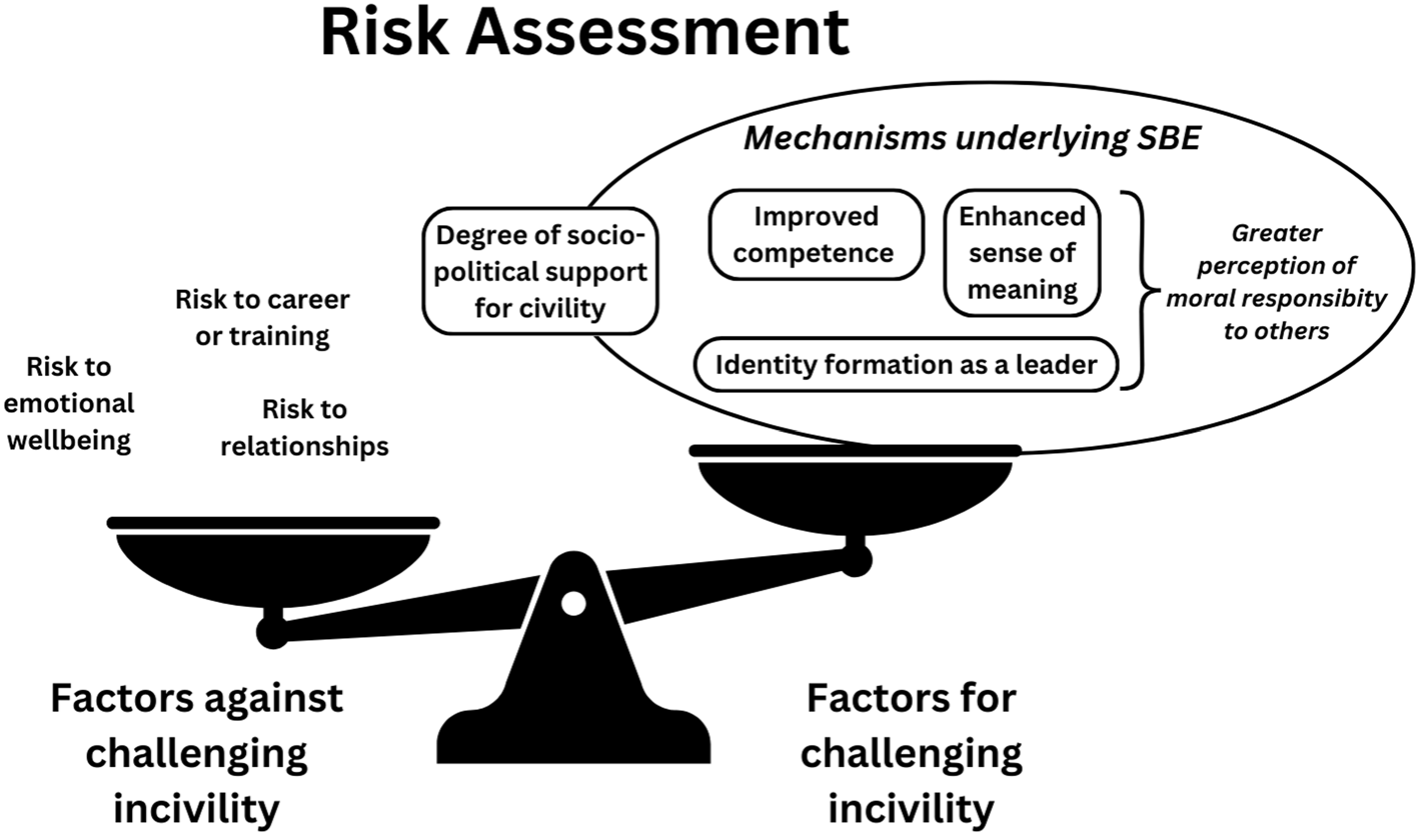
Illustration of the interactions between the factors influencing risk assessment when deciding on response to incivility

Cortina et al. [58] described a theory of biobehavioural responses to workplace incivility, referring to the phenomenon whereby incivility is appraised as a social threat triggering coordinated physiological, emotional, and behavioural reactions aimed at maintaining safety and social belonging. They propose four possible responses to incivility (reciprocation, relationship repair, retreat and recruitment of support) which are mediated by social affiliation between involved parties [58]. This is reflected in the theme of *transfer*, with a sense of social connection and belonging influencing the decision to challenge rudeness. Within this biobehavioural theory, affiliative actions could be directed towards the incivil party (relationship repair) or the social network (seeking support from others after experiencing incivility) [58]. Our findings suggest that this theory could be extended to include a sense of *moral responsibility to others* as an additional affiliative factor that may mediate the response to incivility. We found that immersive SBE focused on relational aspects of work appeared to influence this sense of social connection and moral obligation, ‘shifting the scales’ on benefit versus risk assessment of intervention when incivility occurs (Fig. 3).

In this study, we conceptualise a model for the mechanisms underlying SBE focused on professional relationships, utilising Spreitzer’s theory of psychological empowerment in the workplace with inclusion of socio-structural mediators of transfer to practice. Although evidence exists for SBE influencing domains related to psychological empowerment such as self-efficacy [59–62], this study is novel in the use of psychological empowerment theory as a conceptual framework to understand and evaluate SBE. We explore the mechanisms underlying the influence of SBE using a critical realist lens, uncovering the ‘how and why’ of SBE in this context. For example, in this participant group of medical registrars, identity formation towards a senior leadership role appeared to be an important mechanism underlying the development of self-determination. This occurred within incivility-related SBE through the act of experience sharing with peers with subsequent reflection on the responsibility to others that is integral to the medical registrar role. We propose that these mechanisms underlying different domains of psychological empowerment could have utility in the design, delivery, and evaluation of SBE related to professional behaviour and workplace relationships. Consider a proposed learning outcome that does not hold any meaning to the participant; in this scenario, it is unlikely the educational activity will translate into behavioural change. We might then consider deliberate methods to introduce or consolidate a sense of meaning within the simulation activity, as explored in Table 2. This would be an interesting avenue of future research.

**Table 2:** Potential methods to introduce a sense of meaning within SBE objectives, using incivility as an example

Stage of Simulation Activity	Method to Introduce Meaning
Pre-course	Information sharing raising awareness of the impact of incivility in different accessible options: for example, sharing of “Civility Saves Lives” TED talk or infographic (11)
Scenario design	Highlighting the detrimental impact of incivility through designing and displaying a clear impact on individual performance, team working or interprofessional relationships utilising embedded faculty
Debriefing	Incorporating deliberate debrief discussion points, for example exploring participants experiences of incivility in the workplace, and the impact such experiences had on individuals and teams
Post-course	Sharing of consolidation materials in a different form to pre-course resources, for example a podcast

### Strengths and limitations

This study harnessed theory from business management and organisational development, utilising a critical realist lens to better understand the mechanisms underlying the influence of an incivility-based simulation on subsequent real-world experience. We acknowledge that in utilising psychological empowerment theory, we have chosen an individualistic lens for a topic that is inherently relational and influenced by socio-structural context. This may limit the applicability of our findings; however, we took steps to address this through consideration and incorporation of socio-structural factors within the analysis. We also note that this theory was validated within a business context and therefore may not fully encapsulate the domains of psychological empowerment within healthcare organisations.

We acknowledge that this study focused on the experiences of a medical registrars within South-East Scotland and therefore may not be transferable to healthcare workers in different professional groups, contexts, and geographical locations. Participants also volunteered to join the study and therefore may have been more supportive of the SBE than those who declined. The time gap between the simulation activity and the research interview also varied significantly between participants, which may have influenced responses through either having a limited time frame to encounter incivility or conversely difficulty in recalling experiences or reflections as they relate to SBE. Considering the relatively small sample size in light of Malterud *et al.’s* concept of ‘information power’ [63], we felt that this sample was sufficient to answer the research question given the narrow study aim, specific participant sample, grounding in relevant theory, quality of interview data and detailed analysis resulting in the generation of new knowledge. A valuable avenue for future research is exploration of the feasibility and impact of incivility-focused SBE within interprofessional teams. A further limitation is the reliance on participant recall within interviews regarding experiences of workplace incivility. Given the unpredictability of incivil encounters, an ethnographic approach was not practical, but participant diaries could have complemented our findings.

## Conclusions

This critical realist study highlights the potential role of immersive SBE as a powerful tool for exploring professional interactions and relationships, which may influence behavioural change through stimulating reflection related to the cognitive domains of psychological empowerment. We explore SBE as an influencer of the nuanced risk assessment that individuals undertake when faced with incivility. We propose that this extended framework of psychological empowerment may have utility in designing and evaluating SBE which focuses on relational aspects of work. We encourage all simulation educators to incorporate SBE related to professional interactions into their routine practice, as topics such as managing incivility are relevant to all healthcare professional and interprofessional groups.

## Supplementary Information

Supplementary Material 1.

Supplementary Material 2.

## Data Availability

The datasets used and/or analysed during the current study are available from the corresponding author on reasonable request.

## Abbreviations

ILO: Intended Learning Outcome
IMT: Internal Medicine Training
SBE: Simulation-Based Education
