# Supplementary material for: Education that Empowers: A critical realist evaluation of a novel incivility-focused immersive simulation
Source: Adv Simul (Lond). 2026 Mar 19;11:23. doi: 10.1186/s41077-026-00413-2 (PMC13001293; doi:10.1186/s41077-026-00413-2)
Supplement: Supplementary file 1 — Supplementary Material 1. [file 41077_2026_413_MOESM1_ESM.docx]

**Additional File 1: Incivility Simulation Scenario**

**Title: Med Reg Sim – Civility and Managing a Team**

**Participants:** Doctors working at medical registrar level (IMT 3 – 7)

**Estimated running time:** 15 mins

**Intended learning objectives:**

By the end of the simulated scenario and debrief, learners should be able to:

1. Discuss strategies to challenge incivility with compassion
2. Recognise factors that contribute to incivility
3. Demonstrate strategies to support colleagues combat these factors
4. Demonstrate an approach to managing and resolving conflict within a team

**Case summary/ Story line:**

You are the medical registrar on hospital at night for a district general hospital. The foundation year 2 (FY2) doctor has called you to the MAU doctor’s office to discuss a patient they have seen. When you arrive, you witness the FY2 being rude over the phone to the hospital at night coordinator (advanced nurse practitioner, ANP). The FY2 puts down the phone and turns to you to handover the patient.

**Personnel:**

- **Medical FY2 (in person – the faculty member who knows the participants least well)**
- **HAN ANP - coordinator (over phone)**

**Case summary/ Story line for faculty:**

- This scenario occurs in a non-clinical space and focuses on the participant responding to and managing incivility and conflict within the team. One participant should lead this scenario.
- One embedded faculty member acts within the scenario as the foundation year 2 (FY2) doctor who is overhead by the participant being rude over the phone to the hospital at night (HAN) coordinator. The participant is expected to explore and challenge the incivil interaction, revealing the factors contributing to the incivility.
- One faculty member will act over the telephone as the HAN coordinator. Initially the HAN coordinator will be angry with a risk of conflict within the team. This should resolve as the participant explores and communicates the situation.
- Please see the information within the storyboard states for conversational cues and suggested prompts for embedded faculty.

**Script for embedded faculty prior to participant entering room (for observers to hear)**

HAN coordinator: Hi, it’s the coordinator, [name], again.

FY2: Hi, yeh I’m just in MAU

HAN coordinator: I just have a couple of things for you...oh hold on that’s the phone going again...2 mins...yeh can you hold please, I’m just on the other line...ok I’m back, thanks for waiting

FY2: Ok

HAN coordinator: Ok I have a cannula needing done in ward 21 SR 4, and also they are asking for night sedation. There's also a couple of fluid reviews in MAU in bay 3, beds 1+2 and a gent level that needs to be taken, bay 2 bed 5. And ward 25 have called again about that lady initials MH with a NEWS of 5 but I don’t think much has changed since you last saw her...*(stop talking when participant enters the room at a natural point to allow for response of FY2 in state 1)*

**Appendix 1 Continued: Incivility Simulation Scenario**

**Briefing for candidate:**

You have been called by the medical FY2 to the MAU doctor’s office, as they want to discuss a patient they have reviewed. There are 4 patients waiting to be clerked in A+E, the FY1 is down there but no-one is particularly unwell.

**Location:**

Sim office / non-clinical space

| **EVENTS** | **EMBEDDED FACULTY STATE** | **PARTICIPANT EXPECTED BEHAVIOUR** | **TRANSITION/ notes** | **PROMPT IF REQUIRED** |
| --- | --- | --- | --- | --- |
| **State 1**    **ILO 1:**  Discuss strategies to challenge incivility with compassion | On arrival, participant witnesses FY2 on the phone being rude to the HAN coordinator:  “ugh, am I the only one doing any work tonight? Half this stuff doesn’t even need to be done overnight. They won’t get done at all if you keep bothering me. Don’t you think I have enough to do?! [can swear if feel comfortable]  FY2 slams phone down, sighs and turns to participant  FY2 jobs list on desk - looks very full  [if participant asks about patient query – make it trivial e.g. patient I just clerked, but they are stable. It’s about their morning medications but can wait] | Discuss / challenge incivil behaviour with FY2 | Participant recognises rudeness and holds a conversation with the FY2 regarding the incivil behaviour. | Phone rings (participant to answer):  HAN coordinator (quite angry) “oh, you’re already there. I don’t know if you overheard that, but [FY2 name]’s attitude is appalling, and it’s not the first time tonight. I don’t know what's going on but I need you to sort this out, can you speak to them?”  If participant doesn’t answer phone, FY2 prompts:  “It’s the HAN office, I can’t speak to them again, can you get it?”  FY2 (if not challenging incivility) - “do you think I was rude?” |
| **State 2**    ILO 2:  Recognise factors that contribute to incivility  ILO3:  Demonstrate strategies to support colleagues combat these factors | FY2 opens up with underlying factors:  “I have a million things to do”  “coordinator keeps calling me with more jobs”  “haven’t had a break”  “got hardly any sleep today”  At end of state 2, FY2 leaves to go on a break. If asked about patient – state patient they have clerked is stable and conversation can wait until after break | Participant holds conversation with FY2 exploring factors contributing to incivility and explores strategies to combat these.  May include:   - Encourage to have a break - Review of jobs - prioritisation / delegation - Communication with HAN coordinator about workload | Support and strategies discussed  FY2 reassured, leaves to go for a break | Factors:  “The flat above has building work going on, I hardly got any sleep today”  “I forgot to bring dinner as I woke up late and was in a rush”  Strategies:  “how can I go for a break, I have so much to do!”  “…just need to do this fluid review before I go”  “The HAN coordinator keeps calling me with jobs…I didn’t want to say anything as they already think I’m really slow” |
| **State 3**    ILO 3: Managing team conflict | HAN coordinator calls phone:  “I can’t believe they hung up on me. I’ve half a mind to speak to their supervisor”  “I don’t know why they are acting like this”  HAN coordinator initially angry but calms down with explanation. Reflects that shift has been busy / short-staffed. HAN team covering breaks on wards so tasks fell to FY2 who seemed to be managing ok. | Participant holds a conversation with the HAN coordinator regarding interaction with FY2 and discusses strategies to resolve conflict  May include:   - Active listening – representing both parties points of view - Check in with FY2 once back from break   Strategies to optimise team communication e.g. huddle | Conflict resolved and plan agreed with the HAN coordinator on strategy to manage rest of shift | Initially:  “…no excuse for being rude, I’m stressed too but no-one asks how I’m doing!”  After explanation:  “…I can see why they might have reacted like that; I could have communicated better. I’m worried it will be awkward now…what should I do?” |

**Debrief Discussion points:**

Civility – impact of incivility, circumstances that might lead to incivility in yourself and others, how to challenge with compassion
Support – strategies to recognise and support a colleague in distress

Conflict – what was the approach to managing the team here? Has anyone in similar situations where team has been in conflict? How did this affect the team? Was it resolved – how?

**Signposting resources:**

**NHS Lothian Active Bystander Training:** (search via TUBS)

**NHS Lothian Staff Support Services:** https://www.med.scot.nhs.uk/wellbeing/concerns/health

**Website:** Civility Saves Lives [https://www.civilitysaveslives.co**m/**](https://www.civilitysaveslives.com/)

**Podcast:** <https://podcasts.rcpe.ac.uk/show/clinical-conversations/the-power-of-civility-26-jun-2023/>

| Equipment required |  |
| --- | --- |
| Equipment: | **Where to acquire:** |
| Simulated jobs list | Scenario folder |
| Telephone | Sim suite |
| Chairs x2 | Sim suite |
| Desk | Sim suite |
| Computer | Sim suite |
