# Supplementary material for: Education that Empowers: A critical realist evaluation of a novel incivility-focused immersive simulation
Source: Adv Simul (Lond). 2026 Mar 19;11:23. doi: 10.1186/s41077-026-00413-2 (PMC13001293; doi:10.1186/s41077-026-00413-2)
Supplement: Supplementary file 2 — Supplementary Material 2. [file 41077_2026_413_MOESM2_ESM.docx]

**Additional File 2: Interview Schedule**

**Context**

1. Purpose of interview
   1. Clarify definitions of incivility here
      1. *Incivility can be thought of as rude, dismissive, disrespectful or aggressive behaviour that results in psychological or physiological distress*
2. Check happy with consent process. Highlight option to withdraw at any time without giving a reason
3. Confirm happy for recording and explain anonymity process
4. Trigger warning. Option to pause / stop. Signpost to ability to discuss with researcher / peer support if needed
5. Explain need to avoid any patient or colleague identifiable details
6. Demographics check

**General Experiences**

1. Any experiences before the sim scenario that stick in the mind regarding incivility? What happened?
2. What do you remember about the incivility scenario? [can remind participant if needed]
   1. What do you feel you learned / took away from this scenario at the time?
3. Have you done anything differently since experiencing this sim scenario?
   1. Has this changed the way you think or feel about your role?
   2. Has it influenced your relationships?

**Specific Experiences**

1. Have you had any times where you feel you have been able to put your experience from incivility sim into practice?
   1. What happened?
   2. Why was this an important example for you? How do you think the sim influenced this experience? (enablers)
   3. Any other times? (can prompt individual or witness experiences)
2. Has there been times you have experienced incivility and not felt able to put experience into practice?
   1. What happened?
   2. What do you think stopped you? (barriers)
   3. Any other times?
   4. What would need to happen / be present to be able to put this into practice?
3. Anything else we haven’t discussed that you would like to share?
